# In the Water—Instructions by Which We May Keep from Drowning

**Published:** 1883-03

**Authors:** 


### IN THE WATER.

### INSTRUCTIONS BY WHICH WE MAY KEEP FROM DROWNING.

The human body weighs a pound in the water, and a single chair will carry two grown persons. That is, it will keep the head above water, which is all that is necessary when it is a question of life or death. One finger placed upon a stool or chair or a small box, or a piece of board, will easily keep the head above water, while the two feet and the other hand may be used as paddles to propel toward the shore. It is not at all necessary to know how to swim to be able to keep from drowning in this way. A little experience of the buoyant power of water, and faith in it, is all that is required. We have seen a small boy who could not swim a stroke propel himself back and forth across a deep, wide pond by means of a board that would not sustain five pounds weight. Children and all others should have practice in the sustaining power of water. In nine cases out of ten the knowledge that what will sustain a pound weight is all that is necessary to keep one's head above water will serve better in emergencies than the greatest expertness as a swimmer. A person unfamiliar with the buoyant power of water will naturally try to climb on top of the floating object on which he tries to save himself. If it is large enough that is all right. But it is generally not large enough, and half of a struggling group is often drowned in the desperate scramble of a life and death struggle to climb on top of a piece of wreck, or other floating object, not large enough to keep them all entirely above water. This often happens when pleasure boats capsize. All immediately want to get out of the water on top of the overturned or half-filled boat, and all are drowned except those whom the wrecked craft will wholly bear up. If they would simply trust the water to sustain ninety-nine hundredths of the weight of their bodies, and the disabled boat the other hundredth, they might all be saved under most circumstances. An overturned or water-filled wooden boat will sustain more people in this way than it will carry. It would keep the heads above water of as many people as could get their hands on the gunwale. These are simple facts, easily learned, and may some day save your life.

